# Learning Disabilities in Primary School. How to Diagnose and Remediate the Difficulties with a Team Approach: The First Results

**DOI:** 10.11621/pir.2021.0403

**Published:** 2019-12-24

**Authors:** Janna M. Glozman, Anna Plotnikova

**Affiliations:** a Lomonosov Moscow State University, Moscow, Russia; b Lyceum 109, Ekaterinburg, Russia

**Keywords:** Learning disabilities, primary school, neuropsycho logical assessment, remediation, team approach.

## Abstract

**Background:**

An important problem of our day is the significant increase in the number of learning-disabled pupils all over the world. This has led to the emergence of *a new branch of neuropsychology!— “school neuropsychology”* or the “Neuropsychology of learning.”

**Objective:**

This paper analyzes the role and functions of a neuropsychologist in primary schools and the possibilities of his/her collaboration with other specialists in diagnosing children’s problems and organizing remediation for problematic kids.

**Design:**

We established four steps for launching neuropsychological work at primary schools: 1) setting up a screening group for neuropsychological assessment of all children entering the first year of school; 2) a comprehensive neuropsychological assessment of the children who showed poor results in the first step of the study; 3)ateam remediation program; and 4) evaluation of the remediation results by a new neuropsychological assessment at the end of the remediation program.

**Results:**

The results of the first step of our study showed a very high percentage of children with cognitive problems— 37% of 202 6–8 year-old schoolchildren entering the first year of school. They formed a group at risk for future learning disabilities and maladjustment at school. Age and gender differences, and the structure of cognitive underdevelopment, were discussed in the second step of our study. In the third step, a team of school specialists, including a neuropsychologist, a teacher, a school psychologist, and a school social worker, implemented a remediation program which was created and supervised by a neuropsychologist.

**Conclusion:**

A comprehensive neuropsychological assessment of the pupils revealed a complex structure of cognitive disturbances which interfere with pupils’ learning abilities in primary school. The team approach can efficiently prevent learning disabilities and help children with cognitive underdevelopment and risks of future unsuccess at school, when this collaboration of school specialists has a common theoretical approach and is based upon comprehensive neuropsychological assessment.

## Introduction

A pre-eminent contemporary problem in international psychology and pedagogics today is the significant increase in the number of “practically healthy” children who have problems in learning and adjustment to primary school. Once accepted in elementary school, these children manifest a lack of readiness for schoolwork, and cognitive, emotional, and behavioral disturbances. Different authors have proposed various causes for these learning problems: psychophysiological problems (Bezrukih& Kreshenko, 2004); social maladjustment (Andreeva & Danilova, 2020); giftedness (Pylayeva, 2015); and limited health (Nikolskaya, 1995). Without special psychological and pedagogical assistance, such children become maladjusted to school by the end of the first year. School maladjustment means functioning in a way that does not correspond to the child’s psycho-physiological capacities and needs, nor to the requirements of his/her micro-social environment. It manifests itself in difficulties following school programs, the child failing to perform up to his abilities, and in frequent refusals to attend school and/or to follow its rules (Glozman, 2011, 2017; Prieler et al., 2018; Schwarz & Gawrilow, 2019).

According to various estimates, the number of “problematic” children exceeds 30% of students and ranges from 15% to 40% in primary school (Mikadze, 1998; Sapir & Nitzburg, 1973). Some authors relate it to the socioeconomic situation of single-parent families (Mencarini, Pasqua, & Romiti, 2019).

Problems of self-regulation, up to the level of attention deficit/hyperactivity disorder (ADHD), are a frequent cause of learning disability and of maladjustment to school, because self-regulation has cognitive, behavioral, and emotional influence on a person’s well-being and achievement (Kanfer, Reinecker, & Schmelzer, 2006). “Self-regulation assists the achievement of goals by helping to bridge the gap between intention and behavior.” (Schwarz & Gawrilow, 2019, p. 10)

ADHD has severe social and educational consequences. This syndrome manifests itself mostly in modified characteristics of cognitive processes and attention. The main manifestations of the syndrome are *disturbances of attention concentration,* resulting in instability, low selectivity, distractibility with frequent switches of attention (that is, a lack of a selective, sustained, executive, and orienting attention, strategy generation and use, etc.), and *increased unstructured activity*. The latter is expressed via agitation, fussiness, numerous movements not appropriate for the situation (*i.e*., without purpose and functional significance), the inability to sit still, and talkativeness. In class, a child with ADHD often interrupts teachers; cannot sit still at the desk for a long time, so often drops belongings to move under the desk; can suddenly get up and get out of the class, etc.

According to many Russian and Western researchers, the manifestation of attention-deficit and hyperactivity disorder in pediatric populations has grown exponentially in recent years. While the average number of children diagnosed with ADHD is 7–8% of the entire population, our studies show that the symptoms of ADHD have been observed in the vast majority (81%) of children needing remediation for learning difficulties at school (Glozman, 2013, 2017).

The presence of learning problems is also a risk factor for disturbances in child-parent relations and communication with peers, which in turn aggravates a learning disability. The secondary symptoms of learning disability include *emotional instability*, leading to rapid changes of mood, sometimes from aggression to affection. Disapproval and rejection by others often lead to poor self-esteem and communication difficulties. Especially dangerous for a child’s personal and emotional well-being is the situation where the parents’ negative attitude toward him coincides with the others’ negative and accusatory opinions.

*Emotional problems* have a profound impact on children’s development, behavioral functioning, and physical health. Yet, there remains controversy as to whether or not emotional instability is a part or a consequence of the disorder**.**

A new and interesting approach to learning disability is to explain and overcome it by inducing the child to form a responsible attitude, which is understood to be the capacity to vouch for their actions appropriately and in a way that fits social norms, including: obedience in family settings, accepting their own mistakes, trusting their parents, taking responsibility in the school setting, being friendly and willing to help, and caring for the environment. (Martín-Antón, Carbonero, Valdi vieso, & Mon salvo, 2020). It has been shown that such activity has a direct effect on academic performance and commitment (Carbonero, Martín-Antón, Otero, & Monsalvo, 2017). Positive effects were obtained in the children’s motivation and prosocial behavior, including reduction of violent behaviors, and/or improvements in the classroom climate (Manzano-Sánchez & Valero-Valenzuela, 2019).

Many authors consider it essential to involve the family in the learning process, thus incorporating the process into the child’s everyday life (Belando, Ferriz-Morell, & Moreno, 2012; Camacho, Jiménez-Iglesias, Rivera, Moreno, & Gaspar de Matos, 2020; Dinisman, Andresen, Montserrat, Strózik, & Strózik, 2017; Olhaberry & Santelices, 2013).

Although there are many different approaches, it should be noted that traditional methods of education and psychological assistance for learning-disabled children are not efficient. The work of a school psychologist or speech therapist, as a rule, lacks the general foundation and training for a school program, and fails to increase the child’s level of adaptation and learning abilities. “Various psychological interventions are grounded in tradition and one’s own beliefs or in subjective theories; a psychologist does not always get clear feedback on the effects of an intervention.” (Juriševic, Lazarová, & Gajdošová, 2019)

Therefore, the need for a theoretical foundation of remediative education for learning-disabled pupils inside elementary schools is evident. A necessary condition for it is precise knowledge about the level of cognitive development and zone of proximal development for each student (Akhutyna & Pylaeva, 2003). The best possibility for obtaining it is a neuropsychological assessment (Akhutyna & Pylaeva, 2003; Gaddes, 1981; Glozman, 2013, 2017; Hynd & Obrzut, 1981). This is assured by including school neuropsychologists on the staff of primary schools and the emergence of a new branch of neuropsychology— “school neuropsychology” (Hynd & Obrzut, 1981) or the “Neuropsychology of learning” (Gaddes, 1981).

The neuropsychological approach helps a pupil to adjust to school through identifying his/her partial underdevelopment of some mental functions, mechanisms of learning, and behavioral problems, in such a way as to orient the pedagogical and psychological impact, and to work out an individual program of remediation using the strongest aspects of each child’s development (Mikadze & Korsakova, 1994).

A school neuropsychologist alone cannot realize all the potentials of the neuropsychological approach in the context of the actual organization of education in elementary public schools. After the reorganization and unification of public schools, a great number of pupils (up to 200–250 children) were accepted for the same grade. This complicated the primary diagnostics and put an end to an individualistic approach from the start of schooling. Children with low learning abilities became maladjusted to school from the very beginning and manifested related problems such as abnormal behavior, low self-esteem, poor motivation for learning, and psychosomatic symptoms. The number of learning-disabled children permanently increased, and each school group could count 30–70% of such pupils. This interferes with the teacher’s work and emotional state, makes him/her change their approach to education, and/or creates a syndrome of “professional burnout.” As a result, the quality of the teacher’s work and his/her professional identity suffer.

The best way to solve this problem is the team method of dealing with learning-disabled children’s education and remediation inside the primary school. The *team method means the collaboration of all those responsible for the child’s adjustment at school (the teacher, school psychologist, school neuropsychologist, and social worker), unified by common approach, and oriented to diagnosing the mechanisms of the child’s difficulties, the zone of his/her proximal development, and the possibilities for working out an individual program of remediation and development*. It is also important to assure the participation of parents in the remediation program. This means getting parents to adopt an attentive attitude to their child, follow all advice, participate in developing exercises together with the child, attend lectures for parents on how to understand better the specific features of their child, and participate actively in search of ways to help him or her.

The neuropsychological approach needs detailed knowledge about each child’s level of development, which is provided by a comprehensive neuropsychological assessment (Lebedinsky, 1998). It is also specific to a contemporary public school. First, the neuropsychological methods should permit a face-to-face assessment, and must be easily scored and analyzed. On the other hand, these methods must be individual and sensitive. This task can be achieved in a public school by using *a multistep procedure of assessment—* a principle of “sieves” with a diminution of cells at each step.

*The aim of this paper* is to determine this multistep procedure of assessment and to analyze the possibilities for using a team method based upon the neuropsychological approach in public elementary schools.

## Methods

### Procedure

We established four steps for the neuropsychological work at primary school. This paper analyzes the first three steps in detail. The fourth one (the follow-up after rehabilitation) will be the subject of a new paper.

In the *first step* of our study, we used screening methods that permitted us to determine children who needed further assessment and supervision. The selection methods can be used in public schools and identify a risk group in each school group. A general psychologist without neuropsychological specialization can apply these methods.

In the *second step,* the neuropsychologist assessed the children from the *group of those with risks of learning disabilities* (whose scores in the first step were below normal) using Luria’s battery, which was adapted for children with qualitative and quantitative analysis of the data (Glozman, 2012, 2013; Glozman & Soboleva, 2018). The assessment data helped us to create an individual program of remediation for each child, taking account of the child’s specific problems and his/her zone of proximal development. The second aim of the individual neuropsychological assessment was to differentiate pupils who could receive help at school from those who needed to be sent to special remediation institutions.

In the *third step,* all participants of the team worked out an individual program of remediation for each pupil. The program includes not only methods of neuropsychological remediation but also fairy-tale therapy, game-therapy, family therapy, and special methods of pedagogical and social impact. It was also necessary to provide consultations and help the teacher who works with the children most of the time. A part of the remediation program took place at school; for another part we sent the child to special centers for neuropsychological remediation, because the public school did not have sufficient resources to effect the full remediation program.

In the *fourth step (follow-up),* the neuropsychologist repeated the individual neuropsychological assessment to evaluate the results of the effectuated remediation program. The detailed analysis of these data will be the subject of our next paper.

### Participants

Two hundred and two first grade pupils 6-8 years old from five elementary school groups participated in the group screen for neuropsychological assessment in the first step of our study. In the second step, 75 of them formed the group at risk for learning disabilities and had comprehensive individual neuropsychological assessments done by Luria’s methods, together with structured interviews aimed to determine each child’s personality, motivation for learning, and emotional stability. Followed the remediation program at school, 28 children had individual neuropsychological follow-up during the third and fourth step of our study. The remaining 47 pupils with learning disabilities were recommended to visit special centers for neuropsychological remediation.

Let us describe in more details the **methods** used in the first step of the study. We selected the methods that had been successfully used in previous applied neuropsychological assessments of learning-disabled children, since they were accessible for group testing, sensitive enough to reveal the structure of cognitive problems in children of this age, and significant for learning at school.

1. *A modified “correction” (visual search) test* (Glozman & Soboleva, 2018) to reveal difficulties in concentration and sustaining attention. Within one minute, a child had to find and cross out as many letters identical to the indicated example as possible. After one minute, the instruction changed: the child had to cross out one letter and underline another letter during the second minute.

*Diagnostic criteria:*

- Mastering and following the new instruction;
- Concentration of attention after instruction change.

Difficulties in mastering and following the new instruction manifested in:

– Following the old instruction (crossing out the indicated letter);
– Mixing the old and new instructions;
– Failure to carry out one of the instructions (unrelated actions);
– Making errors in following the instruction (the child underlines wrong letters).

**Figure 1.**
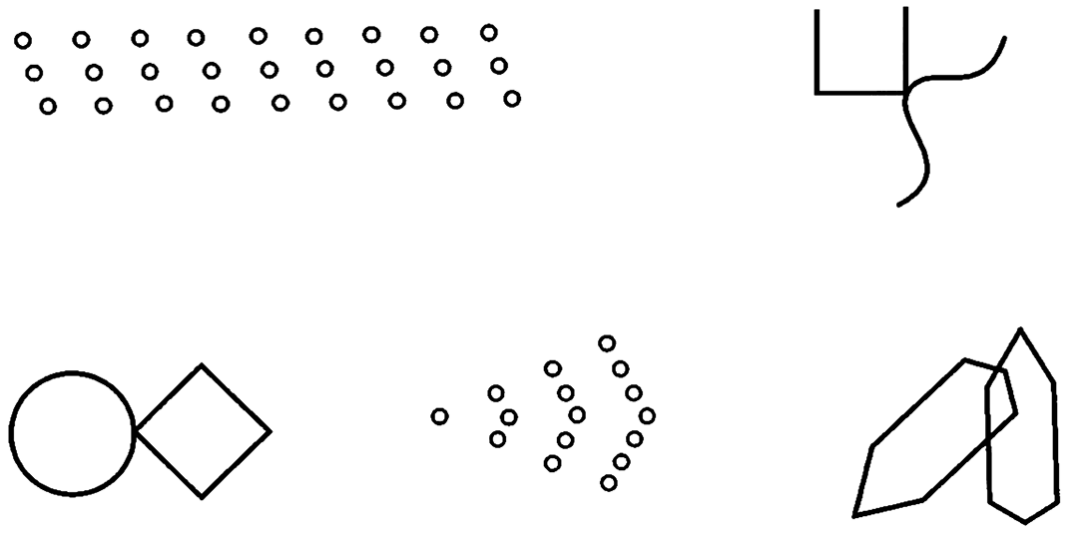
Samples of drawings in the Bender gestalt test

Low concentration of attention manifested in the following statistical criteria:

– Increase in number of mistakes by more than 30% after the instruction change;
– Wrong letters comprised more than 60% of all crossed-out or underlined letters;
– The number of underlined letters (even without mistakes) during the second minute of the test was more than twice lower than during the first minute.

2. *Neuropsychological assessment by a screening group of all children entering the first year of school* (7 school groups) using Luria’s battery, adapted for children with qualitative and quantitative (scoring) analysis of the data (Glozman, 2012, 2013; Glozman & Soboleva, 2018). The assessment aimed to evaluate kinetic, spatial, mnestic, and intellectual functions and included the following tests:

– Graphic test for dynamic praxis;
– Copying a drawing of a table;
– Visual memory test;
– Reasoning tests: a generalization test (which one of four pictures does not belong with the others) and an analogies test.

3. *Bender gestalt test* (Belopolsky, 2003) for evaluation of visual-motor coordination and spatial functions. Each child had to copy as precisely as possible the drawings presented in *Figure 1*. The time was not limited.

Each drawing was evaluated with three scores:

– Right angles;
– Orientation of elements;
– Mutual coincidence of elements.

For each parameter the score varied from 0 to 10, where 0 meant the most precise copying of the example, while the scores 8–10 were given for rotated images; perseverations of the drawings’ elements; gaps exceeding 1 mm between elements of the figure; incomplete figures; inversed angles; and disconnected elements of the figure. The summarized score for all five examples was compared with the table values, differentiated by age (Belopolsky, 2003).

## Results

### Results of the first step of the study

The Thrst important result from analysis of all three parts of the screening group’s neuropsychological assessment of the 202 children entering the first year of school showed that *37% of them (75 pupils) had scores below the normative data (*p < 0.001). We qualified these children as a group at risk for future learning disabilities. Note that their number corresponds to above-mentioned literature data on the frequency of learning problems in primary school.

The *distribution* of the “at-risk children” among different school groups was not equal (*Table 1*). We explain that fact by difficulties in the primary assessment of the children during their admission process for school. Such a distribution can interfere with the efficient organization of education for children with learning problems and successful pedagogical work.

**Table 1:** Distribution of “at-risk children” in different school groups

School group	A	B	C	D	E	F	G
Number/ (%) of “risk children”	10 pupils/ 36%	8 pupils/ 27%	7 pupils/ 23 %	9 pupils/ 31%	13 pupils/ 46%	13 pupils/ 37%	15 pupils/ 50%

Let us now analyze the *gender and age differences*.

Among the 75 pupils from the at-risk group, there were 47 boys and 28 girls. The significant (p < 0.005) predominance of boys correlates with the literature’s data about the greater frequency of abnormal development in boys compared to girls (see details: Mikadze, 1998; Glozman, 2013, 2017).

The age distribution of the group of pupils at risk of learning disabilities was the following: 17 six year-old children, 22 eight year-olds, and 36 seven year-old pupils. An early start to schooling indicated once more the need for early (in preschool age) diagnosis and remediation of delayed development, in order to ensure future successful learning (Shevchenko & Glozman, 2015).

### Results of the second step of the study

**Figure 2.**
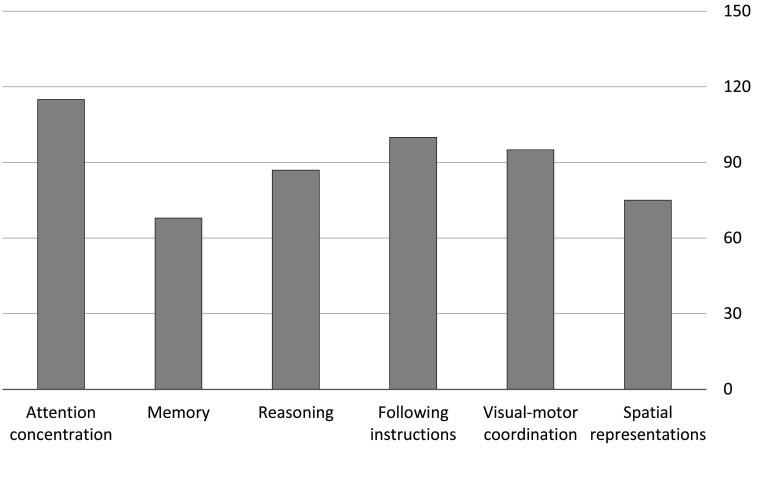
Structure of cognitive disturbances in the first grade pupils (% of children from 75 pupils in the at-risk group)

The most important points for the program of remediation were the data about *the structure of cognitive disturbances* in the first grade pupils at risk of future learning disabilities (*Figure 2*).

We first see a complex structure of cognitive disturbances interfering with pupils’ learning abilities in elementary school. The most pronounced (but not achieving the level of significance) defects were in attention concentration and the ability to follow instructions (neurodynamic and executive functions). These functions assure self-regulation in learning activity, concentration, and the sustaining and distribution of attention necessary for successful learning at school and adequate motivation for learning. They correlate with the data from other studies (see Introduction) on ADHD’s role in learning disabilities in primary school.

### Results of the third step of the study

*The program of remediation* for the children from this group at risk of future learning disabilities took account of the data, as well as of the results of the comprehensive neuropsychological assessment, which revealed the weak and strong components of mental functioning of each child and his/her zone of proximal development. The program included^1^:

1. *Individual neuropsychological remediation*.
  – A course of 20 sessions of neuropsychological remediation carried out by the *school neuropsychologist*. The exercises had to meet each child’s needs and possibilities for realizing the programs inside the public school. The program was carried out in mini-groups of 3–5 pupils.
  – A course of game and fairy-tale therapy, carried out by the *school psychologist,* aimed at stabilizing the child’s emotional state and increasing his/her adjustment abilities.
  – A course of social and pedagogical remediation, carried out by the *school social worker*, aimed at improving the child’s social adjustment, and self-assurance by creating “situations of success.” The number of sessions depended upon each child’s needs.
2. *Preventive intervention in school groups* with a large number of learning- disabled children. The school neuropsychologist worked out this program and effected it together with the school psychologist, working with the group as a whole during gymnastic breaks, walks, pauses between classes, and after class. The program included 30 preventative sessions of neuropsychological and psychological remediation, aimed at developing executive functions, motor functions, and interhemispheric interaction.
3. *The program for teacher support* included:
  – The school neuropsychologist working out a training course for teachers in fundamental principles of the neuropsychological approach to education and some practical methods that the teacher could apply during classes.
  – The school psychologist working out some exercises in art-therapy, relaxation, and elements of psychotherapy in order to help the teacher solve problems of communication with maladjusted children, namely with learning-disabled children.
4. *Lectures by neuropsychologists and psychologists for parents,* which aimed to help them better understand the specific features of their child and to participate actively in his/her remediation.

A collaboration among all specialists included in the child’s education at primary school is mandatory for success of each of these programs. The results of the *fourth step (neuropsychological follow-up)* and the description of the efficiency of this collaboration will be discussed in the continuation of this study.

## Discussion

Our study found a very high percentage of children with cognitive problems and at risk for future learning disabilities and maladjustment at school: 37% of schoolchildren entering the first year of school. This rate correlated with data from many specialists (Akhutina, & Pylayeva, 2003; Glozman, 2017; Mikadze, 1998; Sapir, & Nitzburg, 1973; Schwarz, & Gawrilow, 2019). In this situation, starting school early, at 6 years old, increases the risk of future learning problems. Parents of these children, especially those with pre- and perinatal disorders, should be strongly urged to have a neuropsychological assessment of their child done before his entering school, to confirm that his mental functions (primary neurodynamic and executive) are ready for schooling. If any underdevelopment is revealed, a timely course of neuropsychological remediation could prevent future failure at school.

How to help children already accepted for the first year of school?

1. Delayed cognitive development and its structure must be diagnosed as soon as possible, while learning problems still have not provoked emotional and behavioral consequences. This is now possible due to the inclusion of neuropsychologists on the staff of many big primary schools.
2. Nevertheless, the number of pupils in these big primary schools makes it impossible for one neuropsychologist to assess all of them. Therefore, a two-step assessment procedure is described in detail in this paper:
  – A screening group’s neuropsychological assessment of all children entering the first year of school. A general psychologist without neuropsychological specialization can apply the proposed methods;
  – A comprehensive neuropsychological assessment by the school neuropsychologist of all children who showed poor results in the first step of the study.
2. Such an assessment permits the school neuropsychologist to:
  – Diagnose the structure of cognitive and emotional disturbances interfering with learning abilities for each assessed child;
  – Work out an individualized program of remediation for each pupil;
  – Assure a team approach (participation of the general psychologist, social worker, teacher, and neuropsychologist) in the realization of the remediation program. The paper determines the tasks of each specialist;
  – Reveal in a timely fashion the children who need the help of a specialized remediation institution, because the contemporary public schools all over the world lack the necessary resources to realize neuropsychological remediation in full.

The follow-up of the children who participated in this study can show the efficiency of the proposed team approach for prevention and remediation of learning disabilities

## Conclusion

Neuropsychological assessment revealed the complex structure of cognitive disturbances which interfere with pupils’ learning abilities in primary school. The most pronounced defects were in attention concentration and the ability to follow instructions (neurodynamic and executive functions).

A team approach in public schools can efficiently prevent learning disabilities and help children with cognitive underdevelopment and at risk of future failure at school, when this collaboration among school specialists has a common theoretical approach based upon comprehensive neuropsychological assessment.

The team approach helped realize both diagnostic and remediating tasks, and assured efficient supervision of children with special needs and learning disabilities. A special emphasis should be put on preliminary group assessment of first year schoolchildren to reveal the pupils at risk of future learning problems.

Contemporary public schools lack the necessary resources to realize neuropsychological remediation in full. The main task of the school neuropsychologist is to coordinate the work of other specialists at the school, to work out necessary programs of collaboration, to make a comprehensive individual neuropsychological assessment of each child with learning and behavioral problems, and to make timely decisions revealing which children need the help of a specialized remediation institution.

## Limitations

The findings relate only to the first three steps of our study; they can inform future studies.

## Footnotes

1 This paper does not describe the concrete methods of work, as our main task was to determine the roles of the different specialists in the remediating team
